# Validation of a single liquid chromatography‐tandem mass spectrometry approach for oxytetracycline determination in bull plasma, seminal plasma and urine

**DOI:** 10.1002/dta.3246

**Published:** 2022-03-04

**Authors:** Anisa Bardhi, Teresa Gazzotti, Giampiero Pagliuca, Gaetano Mari, Andrea Barbarossa

**Affiliations:** ^1^ Department of Veterinary Medical Sciences Alma Mater Studiorum—University of Bologna Bologna Italy; ^2^ Health Sciences and Technologies—Interdepartmental Centre for Industrial Research (CIRI‐SDV) Alma Mater Studiorum—University of Bologna Bologna Italy

## Abstract

Oxytetracycline is a broad‐spectrum antibiotic, which inhibits protein synthesis and is generally used for the treatment of pneumonia, shipping fever, leptospirosis and wound infections in cattle and swine. The present work proposes a novel liquid chromatography–tandem mass spectrometry (LC–MS/MS) method for oxytetracycline quantification in bull plasma, seminal plasma and urine, requiring limited sample treatment before analysis. Extraction with trichloroacetic acid followed by dilution of the supernatant in mobile phase proved to be effective in all three matrices, allowing to rapidly process large batches of samples. Sharp and symmetrical peak shape was obtained using a BEH C18 reversed‐phase column in a chromatographic run of just 3.5 min. The mass spectrometer operated in positive electrospray ionization mode and monitored specific transitions for oxytetracycline (461.1 → 425.8) and the internal standard demeclocycline (465.0 → 447.6). The method was validated over concentration ranges suitable for field concentrations of oxytetracycline found in each matrix, showing good linearity during each day of testing (*R* ^2^ always >0.99), as also confirmed by analysis of variance (ANOVA) and lack‐of‐fit tests. Excellent accuracy and precision were demonstrated by calculated bias always within ±15% and CV% below 10% at all quality control (QC) levels in the three matrices. Matrix effect and recovery were investigated for both analytes, which showed consistent and comparable behaviour in each matrix. To our knowledge, this is the first validated approach for mass spectrometric determination of oxytetracycline in seminal plasma and urine. The method was successfully applied to samples collected during a pharmacokinetic study in bulls, allowing to assess the oxytetracycline concentration–time profile in plasma, seminal plasma and urine.

## 1 INTRODUCTION

Tetracyclines (TCs) were first described in the scientific literature and commercialized with clinical success starting from the late 1940s. Due to their broad spectrum of antibacterial activity, easy availability, good treatment efficacy and low costs, TCs are now widely used in both veterinary and human medicine.^1^ To this class belongs oxytetracycline (OTC; molecular formula C_22_H_24_N_2_O_9_, MW 460.4 g/mol), an antibiotic produced by fermentation of certain strains of *Streptomyces rimosus*. This compound is an effective protein synthesis inhibitor in Gram‐positive and Gram‐negative bacteria that targets the 30S ribosomal subunit and specifically impairs the enzyme‐binding of aminoacyl‐t‐RNA to the ribosomal acceptor site.^2^ OTC is generally used for the treatment of pneumonia, transport fever, leptospirosis and wound infections in cattle and pigs.^1^ To study TCs' pharmacokinetic properties, selective and sensitive analytical methods allowing their quantification in different biological matrices are needed. Immunoassay approaches have been described^3^ but with limited specificity and reproducibility. Liquid chromatography–tandem mass spectrometry (LC–MS/MS) is considered the gold standard for drug measurement in biological samples, and several methods have been developed to quantify TCs in human and swine plasma,^4^, ^5^ honey,^6^ muscle,^7^ milk,^8^ shrimps,^9^ medicated feed^10^ and manure.^11^ However, OTC was successfully determined in plasma and seminal plasma by liquid chromatography coupled with photodiode array,^12^ PDA^13^ or UV^14^ detectors. OTC quantification by mass spectrometry has been performed only in plasma.^15^, ^16^ To our knowledge, analytical approaches for its quantification in seminal plasma and urine by LC–MS/MS have not yet been described. The aim of this study was therefore the development and validation of a single method for OTC determination in plasma, seminal plasma or urine by LC–MS/MS. The approach was then applied to samples of three matrices collected during a pharmacokinetic study in bulls.

## 2 MATERIALS AND METHODS

### 2.1 Chemicals and reagents

Analytical standards of OTC hydrochloride and demeclocycline (DEM) hydrochloride were purchased from Toronto Research Chemicals (North York, ON, Canada). Ammonium formate, formic acid, methanol and trichloroacetic acid (all LC–MS grade) were obtained from Sigma‐Aldrich (St. Louis, MO, USA). Ultra‐pure water was produced in‐house (Millipore, Milan, Italy).

### 2.2 Standard solutions

Stock solutions (200 μg/ml) of OTC and DEM (used as internal standard) were prepared by dissolving 5.40 and 5.39 mg, respectively, of pure powder in a 25 ml volumetric flask containing methanol and stored in the dark at −20 ± 2°C. In each day of analysis, fresh working solutions of OTC to be used for calibration and quality control (QC) samples were obtained by serial dilution of the stock solution in water. Similarly, the extraction mixture containing the internal standard at 0.5 μg/ml was prepared by adding 25 μl of DEM stock solution to 10 ml of 10% trichloroacetic acid aqueous solution (pH 2.0).

### 2.3 Sample preparation

The same extraction procedure was applied to plasma, seminal plasma (diluted 1:10 in water) and urine (diluted 1:20 in water), adapting a previously described approach.^5^ An aliquot of 200 μl of each matrix, previously thawed at room temperature, was transferred to a 1.5 ml Eppendorf microtube containing an equal amount of the extraction mixture and 20 μl of water (or of the proper OTC working solution for calibrators and QC samples). The sample was then agitated on vortex mixer for 30 s and centrifuged at 21,000 × *g* for 10 min at 4°C. A 50 μl aliquot of the supernatant was diluted in 450 μl water:methanol 90:10 (*v/v*), with 5 mM ammonium formate and 0.1% formic acid. Extracted samples were kept in the autosampler at 20°C, and 5 μl from each vial was injected in LC–MS/MS.

### 2.4 LC–MS/MS system

The system consisted of a Waters Acquity UHPLC binary pump, equipped with a BEH C18 column (50 × 2.1 mm, 1.7 μm) maintained at 40°C (Waters, Milford, MA, USA). The mobile phase consisted of a mixture of water (A) and methanol:water 95:5 (*v/v*) (B), both with 5 mM ammonium formate and 0.1% formic acid, at a flow rate of 0.3 ml/min under linear gradient conditions.

The LC was interfaced to a Waters Quattro Premier XE triple quadrupole mass spectrometer (Waters, Milford, MA, USA), set in positive electrospray ionization (ESI+) mode with a capillary voltage of 3.00 kV. Source and desolvation temperatures were 120°C and 400°C, respectively; cone gas was set at 100 L/h and desolvation gas at 650 L/h, whereas argon was the collision gas. The detector operated in selected reaction monitoring (SRM) mode: The most abundant transitions for OTC and DEM with their relative cone voltage and collision energy values are reported in Table 1. Data were acquired and processed with MassLynx 4.1 software (Waters, Milford, MA, USA).

**TABLE 1:** Selected mass transitions for oxytetracycline and demeclocycline

Analyte	Transition monitored (*m/z*)	Cone voltage (V)	Collision energy (eV)
Oxytetracycline	461.1 → 425.8	24	20
Demeclocycline	465.0 → 447.6	32	18

### 2.5 Method validation

The method was validated in plasma, seminal plasma and urine using as reference the current European Medicines Agency guideline on bioanalytical method validation during three separated days of testing.^17^ The following parameters were assessed: selectivity, linearity, sensitivity, accuracy, precision, extraction recovery, matrix effect and carry‐over.

During each day of validation, seven‐point (plus a blank) matrix‐matched calibration curves were freshly prepared at optimal concentration ranges (0.02–10 μg/ml for plasma, 0.2–100 μg/ml for seminal plasma and 2–1000 μg/ml for urine) spiking 200 μl aliquots of each matrix with 20 μl of corresponding OTC working solutions. In parallel, QC samples were prepared in triplicates at three different levels, chosen accordingly to each matrix concentration range: 0.05, 0.5 and 5 μg/ml for plasma; 0.5, 5 and 50 μg/ml for seminal plasma; and 5, 50 and 500 μg/ml for urine. Extraction recovery and ion suppression or enhancement for each matrix were evaluated with the method described by Matuszewski et al.^18^ (details are provided as supporting information).

### 2.6 Application to a pharmacokinetic study

The validated method was successfully used for a preliminary study of OTC pharmacokinetic profile in plasma, seminal plasma and urine after administration in one healthy adult bull. The trial was approved by the Animal Welfare Committee of the University of Bologna, Prot. No. 0005783. Samples of blood (in lithium heparin), semen and urine were collected at 0, 12, 24, 36, 48, 72 and 96 h after OTC administration. All the samples were immediately refrigerated, centrifuged for 30 min at 600 × *g* and stored at −80°C until analysis in LC–MS/MS.

During the analysis of each batch of the collected samples, a calibration curve and QC samples were freshly prepared for each matrix as performed for validation, to confirm linearity, accuracy and precision of the method. Drug‐free samples were also injected before and after each series to confirm the absence of carry‐over.

## 3 RESULTS AND DISCUSSION

### 3.1 Method validation

The UHPLC–MS/MS method developed in the present work proved to be the very effective for the quantification of OTC in bull plasma, seminal plasma and urine.

This is not only a single approach that can be applied to three different matrices for OTC quantification but is also, to our knowledge, the first one described for its measurement in seminal plasma and urine by mass spectrometry. The rapid and simple extraction procedure, consisting of protein precipitation and dilution of the sample, is a further strength point of this method, allowing to process 24 samples in less than 20 min. In addition, thanks to the 3.5 min chromatographic run, even large batches of samples can be analysed in a relatively short time. To measure OTC at levels comparable with those found in real seminal plasma and urine samples collected during the pharmacokinetic study, a 10‐fold and 20‐fold dilution in deionized water, respectively, was necessary before sample extraction.

**FIGURE 1:**
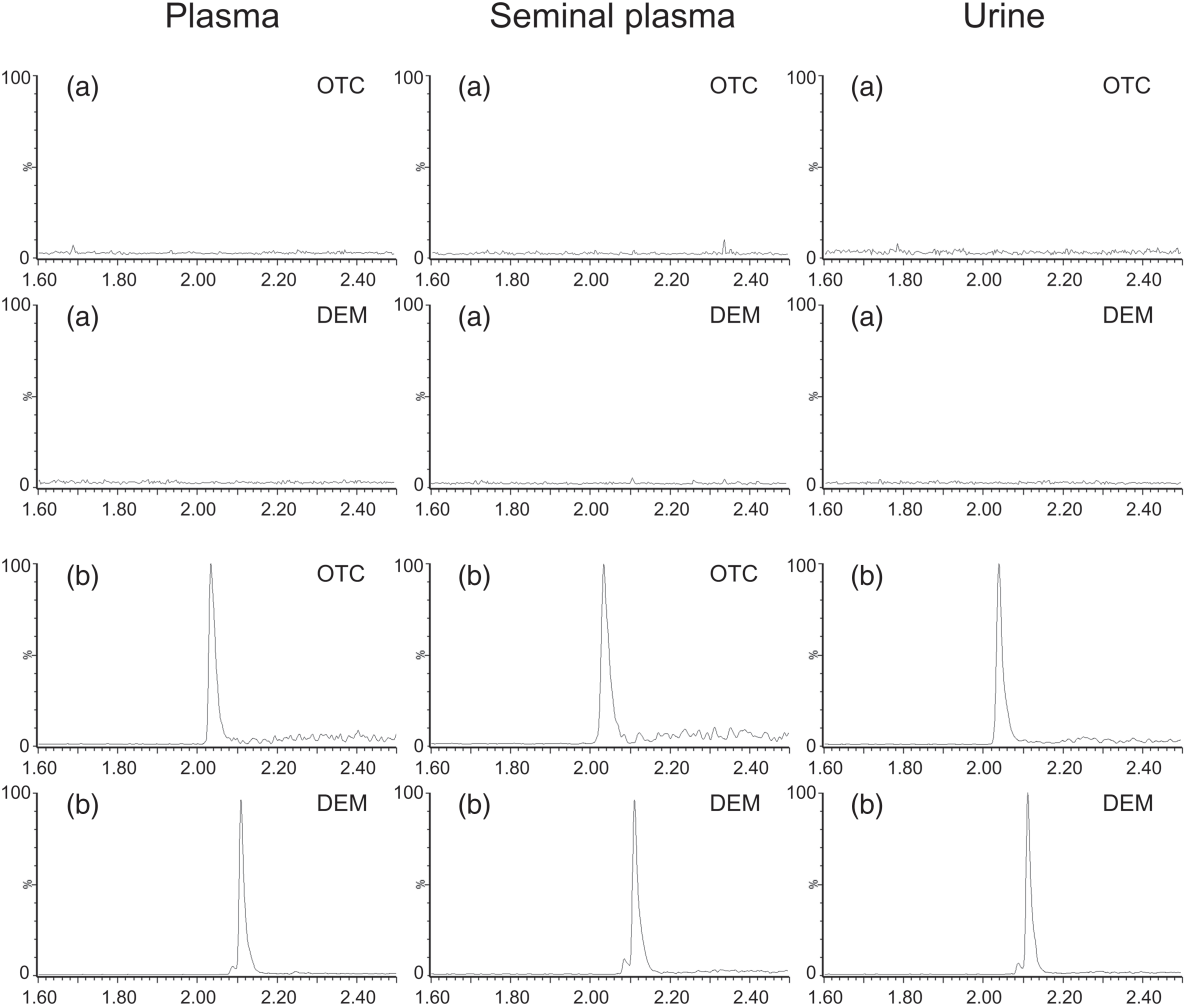
Chromatograms of the monitored transitions of OTC and DEM obtained from the analysis of (a) drug‐free samples and of (b) QC‐low samples of plasma (0.05 μg/ml), seminal plasma (0.5 μg/ml) and urine (5 μg/ml)

The injection of pure standards of OTC and DEM allowed to define their retention time, which was 2.04 and 2.11 min, respectively. The analysis of 10 drug‐free samples for each matrix to confirm the absence of compounds coeluting with the analytes of interest proved the good selectivity of the method, as shown in Figure 1. The resulting coefficient of determination (*R* ^2^) was always ≥0.99 in all the three curves prepared for each matrix. All samples fell within ±15% the nominal value, meeting the pre‐established requirements; moreover, the analytical response of all reinjected samples was within ±15% that of the first injection, indicating the stability of the analyte for at least 12 h at autosampler conditions. Factorial regression (FREG) values, calculated on the three different days of validation, resulted always higher than the tabulated value of 4.60 (*α* = 0.05; df1 = 1; df2 = 14), confirming the validity of the chosen model of regression. The linearity of the calibration curves was also proved by the values generated by the lack‐of‐fit *F* test (LOF),^19^ always below the reported value of 3.58 (*α* = 0.05; df1 = 6; df2 = 8), except for one of the seminal plasma curves, which showed a slightly significant lack of fit. The lower limit of quantification (LLOQ) was 0.02 μg/ml for plasma and 0.2 μg/ml for pre‐diluted seminal plasma and urine.

The results of the accuracy and precision experiments calculated both in repeatability conditions (intraday analysis performed by different operators) and within‐laboratory reproducibility conditions (interday analysis), reported as supporting information, proved the good performances of the method. All QC concentrations tested for each matrix in separate days of validation fell within the ranges established by EMEA/CHMP/EWP/192217/2009 guidelines. In particular, the worst calculated bias of −11.1% was observed in one of the three series of seminal plasma QC‐low samples; similarly, CV% never exceeded 9.0%, which was observed in one of the three series of plasma QC‐low samples.

The ratio between the mean peak area of samples spiked after extraction and that of the corresponding standard calibrators expressed as percentage (values >100% indicate ionization enhancement; values <100% indicate ionization suppression) evidenced, for both OTC and DEM, some ion enhancement in plasma and seminal plasma, whereas a slight overall suppression was observed in urine. The results of the validation, however, suggest that this did not affect the overall performances of the method in either of the two matrices. Internal standard normalized matrix effect was always <15% for the three levels tested in the three matrices, except for the intermediate concentration urine replicates, which showed a CV of 17.0%. This confirmed that DEM is an appropriate internal standard to be used for OTC analysis in biological samples. Average recovery, calculated as percentage ratio between the mean peak area of samples spiked before extraction and the mean peak area of samples spiked post‐extraction, was 82% in plasma, 85% in seminal plasma and 94% in urine for OTC, and 84%, 87% and 99%, respectively, for DEM. In addition, matrix effect (ME) and recovery effect (RE) precision calculated for the five replicates of each type of samples always below 10% (data not shown) proved the absence of subject‐related differences. A complete summary of ME and RE data, as well as the resulting process efficiency values (PE), is presented as supporting information.

### 3.2 Application to a pharmacokinetic study

**FIGURE 2:**
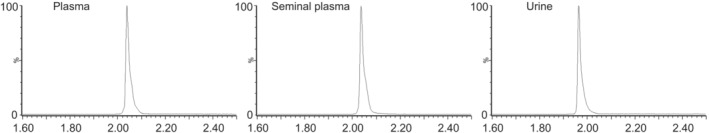
Typical chromatograms for the monitored transition of OTC obtained from the analysis of bull plasma, seminal plasma and urine samples collected during the pharmacokinetic study

**FIGURE 3:**
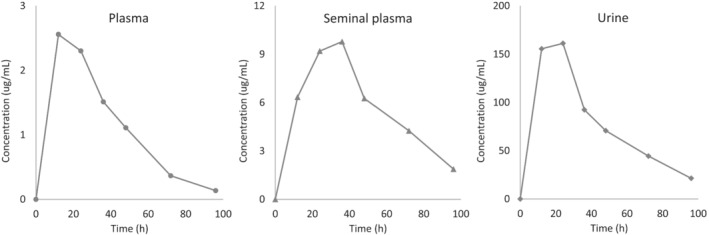
Concentration–time profile of OTC in bull plasma, seminal plasma and urine after subcutaneous administration at 11 mg/kg

The proposed approach was successfully applied to bull plasma, seminal plasma and urine samples collected during a preliminary pharmacokinetic study on OTC, proving that the validated range of concentrations was suitable for the detected levels of the drug (Figure 2 shows chromatograms for OTC obtained processing a real sample of each matrix). The analysis of multiple drug‐free samples during each laboratory session confirmed the absence of carry‐over, even after long series of injections. Concentration versus time curves of OTC in the three matrices are shown in Figure 3: The 4–5 times higher concentration and slower elimination in seminal plasma compared with plasma suggest that this antibiotic could be appropriate to improve the treatment of genital infections in bulls. The present method will be useful for further investigations of the pharmacokinetics of OTC at different dosages, formulations and routes of administration, allowing to optimize therapeutic protocols involving this drug.

## 4 CONCLUSIONS

This work describes for the first time a single LC–MS/MS validated method for the quantification of OTC in bull plasma, seminal plasma and urine. We believe that this approach could be employed not only for research purposes but also in routine clinical laboratory testing involving a still effective and widely used antimicrobial as OTC.

## Supporting information




**Data S1.** Supporting Information

## References

[1] Granados‐Chinchilla F , Rodríguez C . Tetracyclines in food and feeding stuffs: from regulation to analytical methods, bacterial resistance, and environmental and health implications. J Anal Methods Chem. 2017;2017:1315497. doi:10.1155/2017/1315497 28168081 PMC5266830

[2] Nelson ML , Levy SB . The history of the tetracyclines. Ann N Y Acad of Sci. 2011;1241(1):17‐32. doi:10.1111/j.1749-6632.2011.06354.x 22191524

[3] Meyer MT , Bumgarner JE , Varns JL , Daughtridge JV , Thurman EM , Hostetler KA . Use of radioimmunoassay as a screen for antibiotics in confined animal feeding operations and confirmation by liquid chromatography/mass spectrometry. Sci Total Environ. 2000;248(2–3):181‐187. doi:10.1016/S0048-9697(99)00541-0 10805238

[4] Beaudry F , del Castillo JRE . Determination of chlortetracycline in swine plasma by LC‐ESI/MS/MS. Biomed Chromatogr. 2005;19(7):523–528. doi:10.1002/bmc.474 15651016

[5] Bayliss MAJ , Rigdova K , Kyriakides M , et al. Development, validation and application of a novel HPLC‐MS/MS method for the measurement of minocycline in human plasma and urine. J Pharm Biomed Anal. 2019;169:90‐98. doi:10.1016/j.jpba.2019.02.036 30844627

[6] Kivrak İ , Kivrak Ş , Harmandar M . Development of a rapid method for the determination of antibiotic residues in honey using UPLC‐ESI‐MS/MS. Food Sci Technol. 2016;36(1):90–96. doi:10.1590/1678-457x.0037

[7] Gajda A , Posyniak A . Liquid chromatography – tandem mass spectrometry method for the determination of ten tetracycline residues in muscle samples. Bull Vet Inst Pulawy. 2015;59:(3):345–352. doi:10.1515/bvip-2015-0051

[8] Cinquina AL , Longo F , Anastasi G , Giannetti L , Cozzani R . Validation of a high‐performance liquid chromatography method for the determination of oxytetracycline, tetracycline, chlortetracycline and doxycycline in bovine milk and muscle. J Chromatogr A. 2003;987(1‐2):227‐233. doi:10.1016/S0021-9673(02)01446-2 12613816

[9] Reddy BS , Sudhakar Y , Rao YS , Reddyprasad P , Sreedhar NY . LC‐MS/MS Method, Development and Validation to Determine Three Tetracyclines and Their Epimers in Shrimp Samples. Orient J Chem. 2017;33(5):2459‐2469. doi:10.13005/ojc/330539

[10] Gavilán R , Nebot C , Miranda J , et al. Analysis of tetracyclines in medicated feed for food animal production by HPLC‐MS/MS. Antibiotics. 2015;5(1):1. doi:10.3390/antibiotics5010001 PMC4810403 27025516

[11] Loke ML , Jespersen S , Vreeken R , Halling‐Sørensen B , Tjørnelund J . Determination of oxytetracycline and its degradation products by high‐performance liquid chromatography–tandem mass spectrometry in manure‐containing anaerobic test systems. J Chromatogr B. 2003;783(1):11‐23. doi:10.1016/S1570-0232(02)00468-3 12450520

[12] Aktas I , Yarsan E . Pharmacokinetics of conventional and long‐acting oxytetracycline preparations in Kilis goat. Front Vet Sci. 2017;4:1‐5. doi:10.3389/fvets.2017.00229 29312969 PMC5743916

[13] Mileva R , Karadaev M , Fasulkov I , et al. Oxytetracycline pharmacokinetics after intramuscular administration in cows with clinical metritis associated with Trueperella pyogenes infection. Antibiotics. 2020;9(7):392. doi:10.3390/antibiotics9070392 PMC7400317 32659893

[14] Sunaric SM , Denic MS , Bojanić ZZ , Bojanić VV . HPLC method development for determination of doxycycline in human seminal fluid. J Chromatogr B. 2013;939:17‐22. doi:10.1016/j.jchromb.2013.08.035 24095871

[15] Weimann A , Bojesen G , Nielsen P . Analysis of Tetracycline, Oxytetracycline and Chlortetracycline in Plasma Extracts by Electrospray Tandem Mass‐Spectrometry and by Liquid Chromatography. Anal Lett. 1998;31(12):2053–2066. doi:10.1080/00032719808005284

[16] Ziółkowski Z , Grabowski T , Jasiecka A , Zuśka‐Prot M , Barski D , Jaroszewski JJ . Pharmacokinetics of oxytetracycline in broiler chickens following different routes of administration. Vet J. 2016;208:96‐98. doi:10.1016/j.tvjl.2015.08.022 26681141

[17] EMEA/CHMP/EWP/192217/2009 . Guideline on bioanalytical method validation. 2011. https://www.ema.europa.eu/en/documents/scientific-guideline/guideline-bioanalytical-method-validation_en.pdf

[18] Matuszewski BK , Constanzer ML , Chavez‐Eng CM . Strategies for the assessment of matrix effect in quantitative bioanalytical methods based on HPLC‐MS/MS. Anal Chem. 2003;75(13):3019‐3030. doi:10.1021/ac020361s 12964746

[19] Massart DL , Vandeginste BGM , Buydens LCM , De Jong S , Lewi PJ , Smeyers‐Verbeke J . Handbook of Chemometrics and Qualimetrics: Part A. 1st ed. Amsterdam: Elsevier; 1998;1.

