# Supplementary material for: Validation of a single liquid chromatography‐tandem mass spectrometry approach for oxytetracycline determination in bull plasma, seminal plasma and urine
Source: Drug Test Anal. 2022 Mar 4;14(7):1338–42. doi: 10.1002/dta.3246 (PMC9544438; doi:10.1002/dta.3246)
Supplement: Supplementary file 1 — Data S1. Supporting Information [file DTA-14-1338-s001.docx]

**Supplemental material**

**Method validation**

After defining the retention times of oxytetracycline and demeclocycline through the injection of pure standards, the selectivity of the method was assessed analysing blank samples of each matrix from ten different bulls, to verify the absence of chromatographic signals in the same time windows.

During each day of validation, seven-points (plus a blank) matrix-matched calibration curves were freshly prepared at optimal concentration ranges (0.02-10 μg/mL for plasma, 0.2-100 μg/mL for seminal plasma and 2-1.000 μg/mL for urine) spiking 200 μL aliquots of each matrix with 20 μL of corresponding OTC working solutions. In parallel, quality control samples (QCs) were prepared in triplicates at three different levels, chosen accordingly to each matrix concentration range: 0.05, 0.5 and 5 μg/mL for plasma; 0.5, 5 and 50 μg/mL for seminal plasma; 5, 50 and 500 μg/mL for urine. Peak area ratios between oxytetracycline and the internal standard were plotted against their concentration, then a linear regression model was applied. To assess the validity of the regression model, the analysis of variance (ANOVA) and the F-test were performed. The linearity of the analytical response was also investigated by the lack-of-fit test (LOF) [18]. Calibration curves were considered acceptable when the accuracy of 75% of calibrators fell within ±15% the nominal value, except the lower limit of quantification of the curve which should be within ±20% [17]. The lower limit of quantification (LLOQ) of the method was defined as the lowest concentration tested which can be detected with a signal-to-noise (S/N) ratio ≥10 and acceptable accuracy and precision (<15%) in between and within run.

Accuracy, expressed as relative difference between measured value and expected concentration, was evaluated at each QC concentration, and considered acceptable if within ±15% of the nominal value. Precision, defined as the coefficient of variation (CV%) among repeated individual measures, had to be <15% for each QC level. Results are shown in Table SM1.

Immediately after the injection of the highest point of each calibration curve, drug-free samples were analysed to assess the absence of carry-over.

Extraction recovery and ion suppression or enhancement for each matrix were evaluated with the method described by Matuszewski et al. [19]. This approach involves comparing peak areas obtained from three types of samples: A) Standard calibrators in mobile phase, containing the same amount of analytes (OTC and DEM) as QC samples, B) blank samples of each matrix extracted as described above and added with the same amount of analytes, C) samples of each matrix fortified with the same amount of analytes and extracted as described above. Five replicates of each type of samples were prepared, using drug-free matrices collected from five different animals, thus allowing to also assess potential subject-related differences. The matrix effect (ME), sample extraction recovery (RE) and overall process efficiency (PE) were then calculated through comparison of the analytical response of the three types of samples described above, according to the following formulas:

$$ME = \frac{B}{A} (\%)$$

$$RE = \frac{C}{B} (\%)$$

$$PE = \frac{C}{A} (\%)$$

In addition, IS normalised matrix effect was calculated for each type of samples as ratio between analyte ME and internal standard ME, considered acceptable if CV of the five replicates was below 15%. Results are shown in Table SM2.

**Table SM1.** Intra- and inter-day accuracy and precision data obtained for oxytetracycline in bull plasma, seminal plasma and urine at three different QC concentrations in triplicates (n=3) during three separated days of validation.

|  |  | Plasma | |  | Seminal plasma | |  | Urine | |
| --- | --- | --- | --- | --- | --- | --- | --- | --- | --- |
|  |  | Accuracy (%) | Precision (%) |  | Accuracy (%) | Precision (%) |  | Accuracy (%) | Precision (%) |
|  |  | QCL (0.05 μg/mL) | |  | QCL (0.5 μg/mL) | |  | QCL (5 μg/mL) | |
| Day 1 (n=3) |  | -0.3 | 9.0 |  | -11.1 | 8.2 |  | -2.4 | 3.2 |
| Day 2 (n=3) |  | -3.3 | 6.6 |  | 2.2 | 7.8 |  | 2.3 | 1.0 |
| Day 3 (n=3) |  | -10.7 | 3.2 |  | -3.8 | 5.5 |  | 1.8 | 2.5 |
| *Inter-day (n=9)* |  | *-4.8* | *7.7* |  | *-4.2* | *8.7* |  | *0.6* | *3.1* |
|  |  | QCM (0.5 μg/mL) | |  | QCM (5 μg/mL) | |  | QCM (50 μg/mL) | |
| Day 1 (n=3) |  | -0.5 | 2.9 |  | -2.0 | 1.6 |  | 4.2 | 3.6 |
| Day 2 (n=3) |  | 0.8 | 4.0 |  | 2.2 | 3.4 |  | 0.9 | 3.3 |
| Day 3 (n=3) |  | -5.3 | 2.2 |  | 0.9 | 3.9 |  | 2.1 | 4.0 |
| *Inter-day (n=9)* |  | *-1.6* | *3.9* |  | *-0.4* | *3.3* |  | *2.4* | *3.5* |
|  |  | QCH (5 μg/mL) | |  | QCH (50 μg/mL) | |  | QCH (500 μg/mL) | |
| Day 1 (n=3) |  | -1.3 | 1.9 |  | -0.2 | 3.7 |  | -5.2 | 1.5 |
| Day 2 (n=3) |  | -2.6 | 0.9 |  | -2.7 | 1.6 |  | -0.9 | 2.2 |
| Day 3 (n=3) |  | -5.1 | 1.2 |  | -4.3 | 3.0 |  | -3.7 | 4.1 |
| *Inter-day (n=9)* |  | *-3.0* | *2.1* |  | *-2.4* | *3.1* |  | *-3.3* | *3.1* |

**Table SM2.** Results of OTC and DEM Matrix Effect (ME), Recovery (RE) and Process Efficiency (PE) experiments, obtained from five replicates at three spike levels for each type of sample in the three matrices (A = standard calibrators in mobile phase, containing the same amount of analytes as QC samples; B = blank samples of each matrix fortified with the same amount of analytes after extraction; C = samples of each matrix fortified with the same amount of analytes and extracted).

| Conc.  (μg/mL) | Mean peak area  (arbitrary units, ×10^3^, n=5) | | | | | | |  | ME  (%) | |  | RE  (%) | |  | PE  (%) | |
| --- | --- | --- | --- | --- | --- | --- | --- | --- | --- | --- | --- | --- | --- | --- | --- | --- |
|  | OTC | | |  | DEM | | |  | OTC | DEM |  | OTC | DEM |  | OTC | DEM |
|  | A | B | C |  | A | B | C |  |  |  |  |  |  |  |  |  |
| *Plasma* |  |  |  |  |  |  |  |  |  |  |  |  |  |  |  |  |
| 0.05 | 9.7 | 10.3 | 7.9 |  | 80.4 | 93.6 | 74.2 |  | 106 | 116 |  | 76 | 79 |  | 81 | 92 |
| 0.5 | 106.2 | 110.7 | 95.6 |  | 88.2 | 99.5 | 89.3 |  | 104 | 113 |  | 86 | 90 |  | 90 | 101 |
| 5 | 1152.7 | 1132.0 | 946.3 |  | 83.0 | 88.8 | 74.8 |  | 98 | 107 |  | 84 | 84 |  | 82 | 90 |
| Mean |  |  |  |  |  |  |  |  | 103 | 112 |  | 82 | 84 |  | 84 | 95 |
| *Seminal plasma* | | | | | | | | | | | | | | | | |
| 0.5 | 9.6 | 11.3 | 9.3 |  | 87.6 | 99.2 | 90.2 |  | 118 | 113 |  | 83 | 91 |  | 98 | 103 |
| 5 | 113.6 | 119.8 | 109.1 |  | 92.4 | 96.5 | 84.2 |  | 105 | 105 |  | 91 | 87 |  | 96 | 91 |
| 50 | 1034.5 | 1161.7 | 937.5 |  | 99.8 | 103.5 | 86.1 |  | 112 | 104 |  | 81 | 83 |  | 91 | 86 |
| Mean |  |  |  |  |  |  |  |  | 112 | 107 |  | 85 | 87 |  | 95 | 93 |
| *Urine* |  |  |  |  |  |  |  |  |  |  |  |  |  |  |  |  |
| 5 | 45.8 | 43.9 | 43.0 |  | 86.2 | 84.5 | 86.5 |  | 96 | 98 |  | 98 | 102 |  | 94 | 100 |
| 50 | 472.3 | 416.6 | 387.9 |  | 78.7 | 80.8 | 75.8 |  | 88 | 103 |  | 93 | 94 |  | 82 | 96 |
| 500 | 4328.5 | 4003.9 | 3651.5 |  | 81.2 | 76.0 | 77.2 |  | 93 | 94 |  | 91 | 102 |  | 84 | 95 |
| Mean |  |  |  |  |  |  |  |  | 92 | 98 |  | 94 | 99 |  | 87 | 97 |
